# Well-Being Tracking via Smartphone-Measured Activity and Sleep: Cohort Study

**DOI:** 10.2196/mhealth.7820

**Published:** 2017-10-05

**Authors:** Orianna DeMasi, Sidney Feygin, Aluma Dembo, Adrian Aguilera, Benjamin Recht

**Affiliations:** ^1^ Department of Electrical Engineering and Computer Sciences University of California, Berkeley Berkeley, CA United States; ^2^ Berkeley Institute of Data Science University of California, Berkeley Berkeley, CA United States; ^3^ Department of Civil and Environmental Engineering University of California, Berkeley Berkeley, CA United States; ^4^ Department of Agricultural and Resource Economics University of California, Berkeley Berkeley, CA United States; ^5^ School of Social Welfare University of California, Berkeley Berkeley, CA United States; ^6^ Zuckerberg San Francisco General Hospital Department of Psychiatry University of California, San Francisco San Francisco, CA United States

**Keywords:** depression, mobile health, smartphones

## Abstract

**Background:**

Automatically tracking mental well-being could facilitate personalization of treatments for mood disorders such as depression and bipolar disorder. Smartphones present a novel and ubiquitous opportunity to track individuals’ behavior and may be useful for inferring and automatically monitoring mental well-being.

**Objective:**

The aim of this study was to assess the extent to which activity and sleep tracking with a smartphone can be used for monitoring individuals’ mental well-being.

**Methods:**

A cohort of 106 individuals was recruited to install an app on their smartphone that would track their well-being with daily surveys and track their behavior with activity inferences from their phone’s accelerometer data. Of the participants recruited, 53 had sufficient data to infer activity and sleep measures. For this subset of individuals, we related measures of activity and sleep to the individuals’ well-being and used these measures to predict their well-being.

**Results:**

We found that smartphone-measured approximations for daily physical activity were positively correlated with both mood (*P*=.004) and perceived energy level (*P*<.001). Sleep duration was positively correlated with mood (*P*=.02) but not energy. Our measure for sleep disturbance was not found to be significantly related to either mood or energy, which could imply too much noise in the measurement. Models predicting the well-being measures from the activity and sleep measures were found to be significantly better than naive baselines (*P*<.01), despite modest overall improvements.

**Conclusions:**

Measures of activity and sleep inferred from smartphone activity were strongly related to and somewhat predictive of participants’ well-being. Whereas the improvement over naive models was modest, it reaffirms the importance of considering physical activity and sleep for predicting mood and for making automatic mood monitoring a reality.

## Introduction

A goal of personalized medicine is to tailor treatments to individuals based on their needs. To aid the tailoring of treatments, it is necessary to monitor an individual’s state of well-being and to evaluate whether they are responding to a treatment [1,2]. However, monitoring can be a tedious and expensive process and, as a result, can yield low adherence [3]. To overcome low patient adherence, automatic monitoring can be employed in the treatment of mental health disorders, such as depression and bipolar disorder, which benefit from monitoring symptoms over time to identify symptom relapse and to possibly prevent symptoms because of higher self-awareness [4].

The proliferation of personal electronics has enabled continuous personal monitoring [5]. For example, activity recognition has enabled tracking to monitor physical exertion and sleep patterns [6]. Recent studies have started examining whether these smartphone-measured behavioral patterns can be used to infer and then automatically track signals that are not explicitly measured by the smartphone, such as mental well-being.

Many studies have looked at inferring measures of mental well-being from smartphone-measured behavioral patterns [7]. In particular, researchers have considered using measures of location and mobility from global positioning system (GPS) logs to infer depression [8-10], bipolar state [11], stress [12], and well-being measures related to schizophrenia [13]. These studies have shown that daily self-reported levels of stress are related to geospatial activity and sleep [12] and that mobility data can improve predictions of whether a participant is happier or less depressed than usual [8,10] and their bipolar state or transition between states [11]. Researchers have also found that regularity of an individual’s daily mobility is significant when predicting depression symptom severity [9,14].

Additional studies have explored the relationships of social signals such as phone usage, call logs, and SMS (short message service) logs with well-being. Two recent studies found that phone usage measures were correlated with depressive symptom severity [9,14]. Another study found that using social signals such as emails, SMS and call logs, Internet usage, app usage, and location frequency was predictive of mood and energy when previous observations of mood and energy were included [15]. However, a similar follow-up study was unable to reproduce these results. This follow-up study did not find sophisticated models considering high accelerometer activity, call and SMS logs, screen events, app usage, and number of images taken to be better than guessing each individual’s well-being [16].

Whereas this body of literature has established that relationships between measures of mental well-being and smartphone-measured behaviors may exist, the above literature has not focused extensively on physical activity in uncontrolled environments (ie, outside a lab without constraints on participants, such as where the phone must be located). For example, studies have explored predicting bipolar states and state transitions via accelerometers on small populations [11] or mood in constrained environments where the phone had to be in a fixed position [17,18] or activities had to be performed in a lab [19]. One study looked at a measure of total daily physical activity and sleep (as measured with multiple sensors) but within the context of stress and not well-being more broadly, and it did not attempt to predict well-being [12].

Despite these few studies’ limited focus on activity and sleep, there is a body of literature external to mobile health (mHealth) that has established a strong relationship of better mood with increased activity [20-24] and sleep quality [25,26]. There is also mounting evidence that a smartphone accelerometer measures physical activity to a sufficient extent to be useful for monitoring well-being. Several studies have demonstrated that individuals’ sleep and physical activity can be somewhat accurately tracked with smartphones [27] and activity recognition [28-30], respectively. As a result, it seems probable that an individual’s activity and sleep, as tracked by their smartphone’s accelerometer, could be related to and potentially predictive of their mood and well-being more broadly.

If possible, tracking mental well-being with an accelerometer could have benefits over using other sensors. For example, an accelerometer could provide more privacy than previously considered sensors, such as GPS location [8-12] and call logs [13,15,16]. Another advantage to using an accelerometer is that the sensor is always available when the phone is turned on, including when the individual’s phone is out of service or, for example, in a tunnel. Whereas accelerometers embedded in a wearable device might have more potential to accurately track activity, smartphones are more ubiquitous and thus more realistic for long-term tracking.

Here, we are interested in focusing on and better understanding the relationships of physical activity and sleep, as measured by a smartphone accelerometer, with emotion for improving automatic mood tracking. We are particularly interested in understanding whether the relationships are predictive, especially from data collected with ordinary participant-owned smartphones in unconstrained environments (ie, not imposing constraints on participants about where they need to keep the phone or whether they need to have a special device with an accelerometer attached to their body). To explore these research questions, we conducted a field study, extracted measures of physical activity and sleep from smartphone accelerometer logs, related these measures to participants’ self-reported well-being, and attempted to infer participants’ well-being with classification and regression models. We expect that increased physical activity and better sleep quality will be related to improved self-reported mood and well-being.

## Methods

### Field Study

We recruited 106 participants from the university community through the Experimental Social Science Laboratory (XLab) for an 8-week field study to pilot methods. Participants were eligible if they owned an Android smartphone, were native English speakers, were undergraduate students, and agreed to the consent form. The study was approved by the University of California, Berkeley Internal Review Board. The participants were asked to take an entry survey, respond to daily well-being prompts on their smartphone, allow passive collection of sensor data from their smartphone, and take an exit survey.

### Data Collection

Data were collected from participants through a custom Android app that used the Funf Open Sensing Framework [31]. This app was installed by participants before the study period and collected both passive sensor data as well as daily participant input. The participants were instructed and reminded to uninstall the app at study completion.

To quantify well-being, we followed prior studies and asked participants to repeatedly fill out a 2-question survey on their phone. Participants could enter information about their state on two 9-point Likert scales—one for energy and one for mood. Scales were labeled with opposite poles, such as unhappy to happy and unenergetic to energetic. Participants could select the specific words from short lists of relative synonyms for each pole, such as unhappy, negative, sad, bad versus happy, positive, good. Participants were queried for their state 4 times a day. Each of the four daily surveys occurred at a random time within a predefined period between 8 AM and 10 PM. The purpose of randomizing within periods was to ensure distribution of surveys throughout the day without having participants anticipate them. All responses given in a day were averaged into a daily level of perceived mood and energy.

To measure activity, we sampled the smartphone’s accelerometer for intervals of 3 seconds every 5 minutes. These data were collected continuously from the time the app was installed. There were compatibility issues with phone models and network connections, hence, the amount of data collected on each subject varied. Quality of accelerometers also varied between phone models, which contributed to variance in the amount and quality of data collected on each individual. Some of the difficulties we encountered with sensor data collection included entirely missing observations, nonuniform readings during an observation interval, and insufficient duration of sampling, that is, less than 3 seconds. Participants were excluded from the analyses if they did not have complete data (well-being responses and activity readings) for at least 14 days of the study.

### Data Processing

#### Preprocessing

The smartphones’ 3-axis accelerometers measured the acceleration of the device in three directions. Following prior work, we considered the magnitude of the acceleration minus gravity [32]. Gravity for each segment was estimated as the average of coordinates in each of the directions. To account for irregular sampling and to reduce noise in the sensor readings during a sampling interval, we interpolated the available data points and took regular sampling from the interpolation. Quadratic and cubic splines gave irregularities with missing readings; thus, a linear spline was identified as performing the best. This regular sampling allowed us to compute discrete Fourier transforms on the approximated signal and approximate the spectral density using Welch’s method, that is, averaging between Fourier transforms on multiple overlapping segments of the full observation window.

#### Activity Inference

We inferred activity from features summarizing the orientation-invariant magnitude of acceleration deviation and the spectral density of the magnitude of deviation of acceleration. The acceleration deviation was computed by subtracting the estimated gravity from all readings in the interval. This approach was taken to allow for more fine-grained analysis of movement than is presented here. Much prior work with accelerometers, predicting both mental well-being [11,17,19] and activity [28-30], utilized features on coordinate-wise acceleration. However, such approaches were not applicable here, as our participants’ phones were not in a fixed position during the study. We followed prior work that considered features on the magnitude and power spectrum of the magnitude of acceleration during the sample period [30]. The features we used were the average and standard deviation of the magnitude of acceleration and the dominant frequency, entropy of the normalized power spectrum, power in the high frequencies, medium frequencies, and low frequencies of the power spectrum of the magnitude of the acceleration. These eight features were used to fit two logistic regression classifiers. One classifier was trained to identify when the phone is *still* or set down; the second classifier identified *activity* such as walking, running, or pedaling a bicycle. We did not use a classifier to explicitly identify the phone being in a vehicle, such as a car, bus, or train. We did not find a classifier to be reliable enough, given the many states a vehicle can assume, for example, idling, accelerating, and traversing a smooth or bumpy road. Such a task was also of uncertain necessity because participants do not necessarily exert extra energy while riding in transportation and thus vehicle activity was less likely to correspond to elevated mood from physical exertion.. As a result, we focused this study on measures of physical activity and sleep. The goal of these two classifiers was to quantify how long the phone was set down at night, and the subject presumably sleeping, and how long the participant was physically active during the day. These classifiers were trained on an auxiliary activity-labeled dataset that was collected with the same smartphone app and data processing pipeline. The classifiers achieved 80% to 95% accuracy on held out subjects from the training dataset.

### Measure Extraction

#### Sleep Duration

Sleep duration was estimated as the length of the longest period during which the participant was not physically active, starting after 9 PM the prior evening. This period was calculated by looking at the longest contiguous series of observations when the accelerometer data predicted that the participant was *not active* and taking the duration of that period. Whereas this approach likely overestimates the duration of sleep, it should be representative of a period of passivity or evening rest and is preferable to the highly noisy alternative of considering the duration for which the phone was predicted to be still during the evening.

#### Nighttime Stillness

Sleep disturbance, or nighttime stillness, sought to capture sleep disturbance during the time when each participant’s phone was most likely to be set down and the participant presumably asleep, based on their typical behavior. This measure was considered to be the fraction of time that a participant was still during their median period of late evening or when their phone would typically be still, based on their behavior during the study. The period of late evening was defined for each participant by first considering the longest contiguous set of observations during which the phone was predicted to be set down, starting after 9 PM for each day of the study. The median time that this period started, or presumably the phone was set down, for each day of the study defined the beginning of period, and the median time that the contiguous *still* observations ended on each day of the study was considered the end of the period of late evening.

**Table 1:** Daily measures of activity and sleep and how they were calculated.

Type of measure	Measure	How it was measured and calculated
Time	Day of study (semester)	Coded as the number of days since the first day of the study.
	Day of week	Ordinal variable coded Monday (0) through Sunday (6).
Sleep	Sleep duration	Longest contiguous time that the participant was not physically active starting after 9 PM.
Activity	Daytime activity	Fraction of time a participant was physically active during the median active period. The median active period is the time between the median hour the participant became physically active during each day of the study and the median hour that the participant stopped being active during the study.
	Nighttime stillness	Fraction of time the phone was predicted to be still, that is, set down, during the median still period. The median still period was calculated over the course of the study to be the median hour that the longest contiguous still period started and the median hour it stopped.

The nighttime stillness measure for each day of the study was the fraction of observations on that day of the study, which occurred during the late evening period and was predicted to be *still.*

#### Daytime Activity

For a measure of daily physical activity, we consider the daytime activity, which was the fraction of time that a participant was predicted to be physically active during their active period or the period of the day that we would expect each participant to be active, given their typical behavior during the study. The active period of the day was determined by first looking at the longest contiguous set of observations when the phone’s predicted behavior was *not-physically active*, starting after 9 PM. The median time across all the days of the study when this physically not-active period began was considered as the end of the active period, and the median end time of the not-active period was considered the beginning of the participant’s typical active period. The *daytime activity* measure for each day of the study was then the fraction of time that the participant’s phone predicted (with the models discussed previously) that the participant was *physically active* during the participant’s active period.

#### Day of Study

Following prior work, we coded the day of the study as the number of days that had elapsed since the first day of the study [12]. This measure is important to account for potential participant fatigue, and also to represent the progression of the academic semester, which may have had an effect on the participants.

#### Weekday

The day of the week, and thus the potential effect of weekends, was accounted for by coding weekdays with an ordinal variable from 0 to 6, Monday through Sunday (Table 1).

### Analyses

#### Relating Measures to Well-Being

The first set of analyses sought to study the relationship of activity, sleep, and time on daily well-being. To account for the repeated measures design and missing data, we used mixed-effects linear models to relate reported average daily well-being measures to daily behavior measures [33]. We started with a maximal random-effects structure for each well-being measure to allow for individual variation and increase generalizability. Due to lack of initial convergence of the model, we followed suggestions in prior work to look at the covariance of the partially converged model and remove the variable in minimum variance from the random-effects structure [34]. Using this procedure, we removed the measure of sleep disturbance, *nighttime stillness*, from the random-effects structure when modeling mood and removed the scaled ordinal variable coding the day of the week when modeling energy. After this step, both models converged. Activity and sleep measures were centered and normalized within individuals, and time measures were scaled between 0 and 1 before fitting the models to compare the relative sizes of effects.

To ensure the value of the model with maximally justified random-effects structure, we fit two additional models: (1) a model with only random intercepts and no additional random-effects or fixed-effects and (2) a model with fixed-effects and a random intercept only. Model fit was assessed with chi-square tests on the log likelihood values of different models. Model assumptions were visually checked. The linear mixed-effects models and analyses were carried out in the R programming language and environment [35] using the lme4 [36] and lmerTest [37] software packages.

#### Predicting Well-Being

The second set of analyses assessed whether the relationships between daily mood and the activity, behavior, and time features were strong enough to be predictive. To do this, we attempted two tasks. The first task was to predict whether a participant was having a bad day, that is, whether their well-being was lower than their median-reported well-being. Only participants with sufficient observations of each class (at least 5 fine days and 5 bad days) were included in the analysis. The second task was to predict a participant’s level of well-being.

#### Prediction Models

For the first task, predicting whether a participant was having a worse-than-usual day, we used logistic regressions with an L1 and an L2 norm penalty as well as support vector machines (SVMs) and random forests [38,39]. For predicting the daily level of well-being, we used a linear regression model with the elastic net penalty [40] in addition to an Epsilon-Support Vector Regression and random forests. These models were used on individuals’ data to build *personal models*, rather than pooling all individuals’ data into a *global model*. Personal models were used because they have been shown to be the most successful approach to predicting individuals’ responses [13]. Mixed-effects models help to model behavior within the population as a whole while taking into account the fact that individuals have different behavior, but personal linear models are a best-case scenario for predicting individuals’ behavior from their own data.

#### Prediction Framework

For both prediction tasks, we evaluated prediction accuracy with leave-one-out cross-validation on personalized models, that is, we trained a model on all but one of a participant’s data points, evaluated the model accuracy on the held-out observation, and then averaged accuracy across observations. The penalty weights hyperparameters were set with leave-one-out cross-validation on the training data and scanning a variety of penalty weights. The predictive analysis was performed in Python with the scikit-learn library [41].

#### Model Evaluation: User Lift

The accuracy of predicting whether an individual was having a good day was quantified by prediction error or the percentage of observations that were incorrectly predicted. The accuracy of predicting the level of well-being on a given day was quantified by root-mean-square error, which is the square root of the average squared distance of a prediction from the true value. We report the accuracy of predictions compared with the accuracy of predicting each participant to be at their most common state. This measure is called *user lift*; it is the increase in accuracy, or decrease in error, that the model has relative to always predicting an individual to be at their most common state [42]. By comparing a model with each participant’s baseline, user lift reveals how much better a model is doing than guessing a participant to always be at their usual state. We then used permutation tests to assess whether user lift was significantly positive across the participants, that is, whether the models were significantly better than always guessing a participant to be at their most common state, as permutation tests are reported to be more reliable than paired nonparametric tests [43,44].

## Results

### Participation

Of the 106 participants recruited, 87 installed our app; 57 completed the study, that is, completed the exit survey at the end of the 8-week study period. However, there were only sufficient data on 53 participants to include in the analyses. Baseline characteristics of individuals included and excluded from the analyses are shown in Table 2 and indicate that similar populations were included and excluded from the analyses. Whereas some attrition was because of participation waning over the 8-week study period, there was also attrition as a result of technical difficulties and app compatibility issues on older phones.

**Table 2:** Participant baseline characteristics. Averages across individuals are reported with standard deviations in parenthesis, except where indicated. Where appropriate, numbers represent the average across individuals of averages within individuals.

Participant measure	Included participants with exit survey (n=47)	Included participants with no exit survey (n=6)	Excluded participants because of insufficient data (n=53)
Age^a^	19.83 (1.99)	20.33 (1.60)	20.80 (4.13)
Female (number)^a^	26	3	28
BDI-20^b^ score (entry)^a^	11.14 (9.27)	7.33 (3.54)	12.61 (7.20)
BDI-20^b^ score (exit)^a^	11.98 (12.00)	N/A	N/A
Median mood rating	5.17 (1.63)	5.83 (0.90)	5.44 (1.44)
Median energy rating	5.60 (1.27)	6.67 (0.94)	5.98 (0.80)
Number of emotion surveys completed	160.51 (44.42)	139.33 (55.01)	30.25 (50.97)
Number of days with emotion ratings	49.45 (8.27)	44.00 (11.06)	10.49 (15.99)
Reported typical sleep duration in hours (from exit survey)^a^	6.88 (1.35)	N/A	N/A
Average duration of inactive period in hours (sensed *sleep duration*)	8.79 (1.22)	8.56 (0.48)	N/A
Number of times per month a participant exercised (from exit survey)^a^	4.24 (5.04)	N/A	N/A
Average minutes active per day (sensed *daytime activity*)	118.78 (32.67)	151.25 (59.68)	N/A
Number of days with sensed activity and mood input	38.60 (9.15)	40.00 (9.64)	3.36 (5.15)

^a^Indicates measures averaged only over submitted responses, as entry and exit survey questions were optional.

^b^BDI-20 indicates optional self-reports to 20 questions of the Beck’s Depression Inventory (the question related to suicidal ideation was omitted).

**Table 3:** Results of fixed-effects for linear mixed-effects model of mood level from smartphone-measured and time variables. The measure for nighttime stillness was excluded from the otherwise maximal random-effects structure.

Fixed-effect	Estimate	Standard error	*t* value (degrees of freedom)	*P* value
Mean mood (intercept)	5.056	0.174	28.973 (49.0)	<.001
Day of study (semester)	−0.059	0.261	−0.226 (47.0)	.82
Day of week (coded 0-6, Monday-Sunday)	0.040	0.076	0.528 (257.0)	.60
Sleep duration	0.072	0.030	2.451 (52.0)	.02
Daytime activity	0.097	0.032	3.062 (50.4)	.004
Nighttime stillness	0.040	0.026	1.528 (1881.5)	.13

**Table 4:** Checking model fits for linear mixed-effects model of mood.

Model name	Akaike information criterion	Bayesian information criterion	Log likelihood	Chi-square value (degrees of freedom)	*P* value
Random intercept only	6522.0	6538.8	−3258.0		
Fixed-effects with random intercept only	6508.8	6553.7	−3246.4	23.2 (5)	<.001
Maximal random-effects structure	6322.0	6445.4	−3139.0	214.8 (14)	<.001

### Relationship of Sensor Data With Well-Being

From linear mixed-effects models, we found significant positive relationships of daytime activity and sleep duration with daily mood; when participants get more sleep and more daily activity they tend to report better moods (Table 3). Daytime activity has a stronger relative effect than sleep duration. Of note is that nighttime stillness (sleep disturbance) is not significant. This lack of significance could imply that the measurement is too noisy and that more work is needed to reliably measure sleep disturbance with a smartphone. The model with the maximal random-effects structure better accounted for the variance across individual participants than the random intercept only model (Table 4). The main effects also remained significant, even when accounting for individual differences.

We also found a significant positive relationship of daytime activity with daily perceived energy level (Table 5). The relation for sleep, though negative, is not significant, revealing a potentially different relationship between the two emotions (mood and energy) with sleep.

Day of the week has a significant positive fixed-effect but had to be removed from the random-effects structure following prior suggestions about how to handle lack of model convergence [33].

**Table 5:** Fixed-effects for a mixed-effects linear model relating daily energy level from smartphone-measured and time variables. The ordinal variable for weekday was excluded from the near-maximal random-effects structure.

Fixed-effect	Estimate	Standard error	*t* value (degrees of freedom)	*P* value
Mean energy (intercept)	5.686	0.184	30.857 (53.9)	<.001
Day of study (semester)	−0.304	0.233	−1.303 (49.4)	.20
Day of week (coded 0-6, Monday-Sunday)	0.196	0.067	2.912 (1876.2)	.004
Sleep duration	−0.027	0.031	−0.858 (57.7)	.39
Daytime activity	0.182	0.039	4.673 (49.6)	<.001
Nighttime stillness	0.024	0.030	0.810 (50.4)	.42

**Table 6:** Checking model fits for linear mixed-effects model of energy.

Model name	Akaike information criterion	Bayesian information criterion	Log likelihood	Chi-square value (degrees of freedom)	*P* value
Random intercept only	6284.2	6301.0	−3139.1		
Fixed-effects with random intercept only	6196.1	6240.9	−3090.0	98.1 (5)	<.001
Maximal random-effects structure	5972.5	6095.9	−2964.2	251.6 (14)	<.001

This effect for day of the week indicated that participants collectively felt more energy at the end of the week, and there is not sufficient evidence to support the idea that weekday affected participants differently.When we changed the variable encoding weekday to a binary variable indicating a fixed weekend of Saturday and Sunday versus the rest of the week, as has been suggested in related work [14], this relationship did not remain significant. An interaction term between a weekend indicator and daily activity was similarly not found to be significant. This lack of significance as a binary variable could be a result of weekends being less defined in our undergraduate population, some of whom may or may not have classes on Friday and thus have had extended *weekends*. The lack of significance could alternatively result from insufficient observations of weekends for each participant. Again, sleep disturbance is not significant, further indicating that there might be too much noise in the variable measuring sleep quality. The model with the maximally justified random-effects structure accounted for significantly more variation across participants than having only a random intercept (Table 6).

### Predicting Well-Being From Sensor Data

The activity, sleep, and time measures described above were also used to predict daily well-being scores. Whereas mixed-effects models were used to understand relationships of activity and sleep measures with well-being within the population, personal models (linear and nonlinear) were used as a maximally personalized and thus somewhat best-case approach for predicting individuals’ well-being [13]. The *user lift*, or improvement of model predictions over a baseline is reported (Table 7). The user lift is the increase in accuracy (or decrease in error) that a model has relative to always predicting an individual to be at their most common state. User lift compares a model’s accuracy with a participant’s baseline; thus, it quantifies how much better a model is performing than the most reasonable constant prediction for each participant.

In general, it was difficult to predict individuals’ well-being on a daily basis with the given information only being about their activity and sleep (Table 7). On average, the best models were able to improve prediction of good and bad mood and energy by 5.44% and 4.92%, respectively. The model prediction performance presented in Table 7 is for linear models (penalized logistic regression and elastic-net penalized linear regression), as those models were found to return higher accuracy than the nonlinear SVMs and random forests. Whereas there was considerable variation in predictability across individuals, permutation tests reveal that user lift was significantly greater than 0, that is, the models were better than naively always predicting each participant to always be at their most common state.

**Table 7:** Statistics on linear models predicting daily well-being from activity measures. Whereas the models provide an improvement overall, there is a range in the ability to model individuals. The *P* values are for permutation tests, checking whether user lift is greater than 0, that is, whether models are significantly more accurate than always predicting each individual to be at their most frequent state.

Problem (model)	Well-being measure	Average user lift	Minimum user lift	Maximum user lift	*P* value
Good or bad day (penalized logistic regression)	Mood (Prediction error)	5.44%	−21.74%	35.00%	.001
	Energy (Prediction error)	4.92%	−22.73%	39.39%	.008
Daily average (linear regression with elastic net)	Mood (RMSE^a^)	0.026	−0.232	0.48	.08
	Energy (RMSE)	0.048	−0.169	0.575	.01

^a^RMSE: root-mean-square error.

## Discussion

### Principal Findings

We found that increased daily activity, as tracked with a smartphone’s accelerometer, positively correlated with participant-reported mental well-being over time. Whereas a positive correlation of activity and well-being has been substantiated in literature external to mHealth [20-24], we have shown that smartphones measure individuals’ daily activity to a sufficient level of accuracy to measure this relationship in everyday life. Although the potential for this result has been shown in environments where constraints were placed on the participants [11,17-19], we found this relationship present when no constraints were placed on participants. Previous work did not find a significant correlation of the total activity in a 24-hour day with stress [12], which could indicate the need for distinguishing daytime activity from nighttime activity, as we have done, or indicate that physical behavior has unique effects on different emotions, which we have observed by considering mood and energy separately.

We also found that a simple measure of sleep duration derived solely from accelerometer data was significantly positively correlated with mood. However, it was not significantly correlated with perceived energy, which supports the idea that there are different relationships between different emotions and physical behaviors. We did not find a significant correlation of either mood or energy with our measure of smartphone-measured sleep disturbance. This may imply that the measure did not sufficiently describe sleep quality and that more work is needed to monitor sleep quality in a sustainable manner. It is possible that a more sophisticated method for predicting sleep, such as the method found in prior works, would allow for a finer measure of sleep disturbance [27].

When we used the activity, sleep, and time measures to predict individuals’ well-being, we found modest but significant improvement over naive baseline models. It is important to emphasize that there was a range in our ability to predict individuals’ well-being from their activity and sleep behavior. This range highlights the need for tracking approaches that tailor to the user. However, it is unclear whether this effect is the result of a range in how thoughtfully individuals responded with their state, phone usage, data quality and quantity, or the strength of well-being and activity relationship between individuals.

### Limitations

A limitation of this study is that participants’ self-reported well-being is subjective, and the population was not clinically assessed. However, the measures of well-being that we used have been widely used and prior research has found simple single-scale measures to be related to longer clinical assessments [45]. Whereas a better measure of well-being could be a longer survey, such a measure would incur significant participant fatigue and likely decrease the duration of participation.

Whether all of the participants’ relevant activity was tracked with smartphones during the study is another concern. There are limitations to activity recognition, especially when the smartphone is not in a fixed position, a participant is performing a nonstandard activity, or the phone is set down, for example, left in a gym locker. However, the study cohort retrospectively reported little vigorous exercise during the study period (Table 2); thus, the underestimation of vigorous exercise is likely to be minor. Such limitations could possibly be partially mitigated with location tracking, but time at a location is not necessarily representative of activity, and poor GPS sensitivity would remain a challenge. Wearables may provide a better facsimile of an individual’s behavior when they are worn, but they have notorious compliance limitations that smartphones do not suffer.

Another limitation was the sample size and lack of clinical population. Some of the individuals in our study cohort did report elevated levels of depressive symptoms in the entry and exit survey. However, the cohort is not necessarily representative of a population with clinically diagnosed mood disorders. Depressed individuals often are less active than the general population, but even small increases in physical activity can improve symptoms [46].

### Conclusions

This study examined the extent to which smartphones’ accelerometers can contribute to passively tracking individuals’ mental well-being in everyday life. We have found that smartphones measure activity and sleep with sufficient accuracy to reproduce prior findings of significant relationships between activity and sleep with mood. Whereas models have a modest, though significant, improvement over naive baseline models in general, the range in predictive capability implies that more work is needed to tailor mood- and depression-tracking apps to individuals.

Our results support the promise for smartphones to be used in sophisticated and long-term monitoring of patients’ well-being. Because smartphone use is high and their presence ubiquitous, the ability to use a smartphone for tracking mental well-being could have a huge impact on mental health care. Smartphone monitoring may improve self-management via smartphone apps, thereby making care more affordable and thus accessible to individuals who currently do not have access to care. Passive monitoring could also be used as an adjunct to clinician-led treatment, thus increasing the quality of care and personalizing treatments.

## Abbreviations

BDI: Beck’s Depression Inventory
BIDS: Berkeley Institute of Data Science
DARPA: Defense Advanced Research Projects Agency
GPS: global positioning system
mHealth: mobile health
RMSE: root-mean-square error
SMS: short message service
SVM: support vector machines
